# Diagnostic value of bronchoalveolar lavage fluid cytokine and lymphocyte profiles for lower respiratory tract infections in patients with lymphoma

**DOI:** 10.3389/fmed.2026.1904717

**Published:** 2026-09-23

**Authors:** Tianqi Qi, Wenming Hua, Jinling Liu, Tao Zhang, Manyu Li, Qi Hao, Xinyue Liu

**Affiliations:** 1 Department of Clinical Laboratory, Aerospace Center Hospital, Beijing, China; 2 Department of Hematology Laboratory, Aerospace Center Hospital, Beijing, China; 3 Department of Respiratory and Critical Care Medicine, Aerospace Center Hospital, Beijing, China

**Keywords:** bronchoalveolar lavage fluid, cytokines, lower respiratory tract infections, lymphocytes, lymphoma

## Abstract

**Objective:**

Distinguishing lower respiratory tract infections (LRTIs) from noninfectious pulmonary infiltrates (NPIs) in patients with lymphoma is challenging. We assessed the diagnostic value of bronchoalveolar lavage fluid (BALF) cytokine and lymphocyte profiles (CPs and LPs).

**Methods:**

This retrospective study included 147 participants: 55 lymphoma patients with LRTIs, 52 with NPIs, and 40 non-lymphoma patients with LRTIs. Fourteen BALF cytokines and ten BALF and peripheral blood lymphocyte measures were assessed by multiparameter flow cytometry. LASSO-selected logistic regression models were developed using five-fold cross-validation. BALF models incorporated CPs and LPs, whereas Base models used routine inflammatory markers, with or without sputum production. Models were compared using DeLong tests, NRI, IDI, and 1,000-resample bootstrap optimism correction.

**Results:**

LASSO retained BALF IL-8 and CD3 + CD4 + for BALF models and CRP and SAA for Base models. Test-set AUCs were 0.845 and 0.853 for BALF-A and BALF-B, vs. 0.973 and 0.969 for Base-A and Base-B, respectively. Base models significantly outperformed corresponding BALF models. Optimism-corrected AUCs were 0.872 for both BALF models and 0.980 and 0.977 for Base-A and Base-B. Adding sputum production improved BALF-model reclassification without significantly increasing AUC and provided no benefit to Base models. Eight immune parameters differed by lymphoma status among patients with LRTIs, with additional infection-type-specific patterns.

**Conclusions:**

BALF IL-8/CD4-based models distinguished LRTIs from NPIs in patients with lymphoma, although CRP/SAA-based models performed better. BALF immune profiling may complement conventional biomarkers and reveal immune signatures associated with lymphoma status and infection type.

## Introduction

Lymphoma, a malignancy arising from lymphocytes, is frequently complicated by infection. Lower respiratory tract infections (LRTIs) are particularly common in patients with lymphoma, largely because of disease- and treatment-related immunosuppression (1). Prompt recognition and management are essential, as severe infections may cause additional complications and increase mortality risk (2). However, diagnosing LRTIs in patients with lymphoma remains challenging for two main reasons (3). First, high-resolution computed tomography findings of chemotherapy-induced lung injury often overlap with those of LRTIs, and mixed infections further complicate etiological diagnosis. Second, conventional cultures of respiratory specimens detect a limited pathogen range, with slow turnaround and low sensitivity. Moreover, immunosuppression may cause infected lymphoma patients to present with mild or absent fever and normal serum inflammatory markers, further obscuring diagnosis (4). Lymphoma and its treatment may also alter systemic and pulmonary immune-cell profiles; characterizing these alterations could improve our understanding of immune dysregulation in patients with lymphoma and LRTIs.

Bronchoalveolar lavage fluid (BALF), sampled directly from the affected pulmonary segment, reflects local inflammatory and immune responses within the lung. Compared with sputum, BALF provides a more direct assessment of the pulmonary microenvironment, particularly when clinical and radiological findings are insufficient for a definitive diagnosis. Analysis of its cellular and particulate components, including resident and recruited immune cells, together with soluble constituents such as immunoglobulins, enzymes, and antimicrobial peptides and proteins, provides deeper insight into the local immune milieu and may help distinguish LRTIs from noninfectious pulmonary infiltrates (NPIs) (5, 6).

In recent years, cytokine profiles (CPs) and lymphocyte profiles (LPs) have emerged as potential biomarkers of infection (7). In lymphoma patients, conventional inflammatory markers remain valuable because they are widely available, routinely measured, and often distinguish infectious from noninfectious conditions. However, they mainly reflect systemic inflammatory burden and offer limited insight into immune mechanisms. CPs and LPs may provide complementary diagnostic information, especially in atypical presentations. More importantly, many cytokines and T-cell subsets are directly involved in LRTIs pathogenesis (8), so they may better reflect disease pathophysiology and represent potential therapeutic targets (9). A distinct BALF signature comprising elevated IL-8 and IL-13 and increased neutrophils distinguished nontuberculous mycobacterial pulmonary disease from noninfectious granulomatous lung disorders (10). In non-Hodgkin lymphoma, IL-6 and IL-8 outperformed fever, procalcitonin (PCT), and CRP for early detection of respiratory bacterial infection, particularly in minimally symptomatic patients (2). Moreover, single-cell transcriptomics identified a CXCL8 (IL-8)-neutrophil recruitment axis as a key determinant of pneumonia severity (11).

Previous studies have primarily focused on analyzing CPs and LPs in peripheral blood (PB) for LRTIs diagnosis. However, lower respiratory tract specimens obtained directly from the infection site offer distinct advantages in differentiating LRTIs, making the analysis of CPs and LPs in BALF essential. Our analyses addressed two distinct clinical and biological questions. The primary analysis compared lymphoma patients with LRTIs (Lym + LRTIs) versus those with noninfectious pulmonary infiltrates (Lym + NPIs), thereby addressing a clinically relevant differential diagnosis in lymphoma patients presenting with pulmonary infiltrates, in whom infectious and noninfectious etiologies must be distinguished. In contrast, the secondary analysis focused on patients with confirmed LRTIs and compared pulmonary immune profiles according to lymphoma status.

## Methods

### Patient screening and enrollment

The inclusion criteria were as follows: (1) age ≥ 18 years; (2) a clinical diagnosis of lymphoma based on the 5th edition of the WHO Classification of Haematolymphoid Tumors; (3) fulfillment of the diagnostic criteria for LRTIs as outlined by the Chinese Thoracic Society (CTS) in 2016 and 2018 (Supplementary Table S1), confirmed by at least two pulmonologists and one microbiologist; (4) provision of informed consent and voluntary participation in the study; and (5) availability of complete data on cytokine levels and lymphocyte subsets. Exclusion criteria included: (1) diagnosis of malignant solid tumors, other hematologic diseases, or autoimmune disorders; (2) history of major solid organ transplantation; and (3) diseases other than lymphoma requiring prolonged use of specific medications, including anti-TNF-α agents, anti-IL-6 agents, Cyclosporine, Tacrolimus, Azathioprine, 6-Mercaptopurine, Mycophenolate mofetil, Sirolimus, Mizoribine, and Hydroxychloroquine. This was a retrospective observational study. Demographics, symptoms, comorbidities, clinical scores, routine laboratory findings, and etiological data were reviewed through electronic medical records. The specimens were collected from patients who underwent bronchoscopy as part of routine clinical care. The protocol was approved by the Ethics Committee of Aerospace Center Hospital (approval no. 2023070) on May 9, 2023. Written informed consent was obtained retrospectively at the time of approval; for deceased or lost-to-follow-up patients, the requirement was waived by the Ethics Committee with prior anonymization and de-identification.

### Bronchoalveolar lavage procedure and fluid collection

BALF was collected before antibiotic therapy or 48∼72 h after discontinuing prior antibiotics. In most cases, bronchoalveolar lavage was targeted toward affected lung segments as noted on chest computed tomography and performed with 50 mL of 0.9% sterile normal saline per lavage. Usually, three lavages were performed, with greater than or equal to 30% of the total lavage fluid being retrieved through gentle suction and filtered through sterile gauze to remove mucus strands (12). We conducted etiological examination on the collected BALF, including smear staining (Gram staining, Ziehl-Neelsen staining, and Periodic-acid silver methenamine staining), cultures (for bacteria, fungi, and mycobacteria), respiratory virus PCR (for *SARS-CoV-2*, *Influenza A*, *Influenza B*, *Respiratory syncytial virus* (RSV), *Adenovirus*, *Human rhinovirus*, *Cytomegalovirus* (CMV), *Epstein–Barr virus* (EBV), and *Mycoplasma pneumoniae* (MP)), and metagenomic next-generation sequencing (mNGS). The remaining BALF samples were centrifuged, and the supernatants and precipitates were separated for cytokines and lymphocyte subsets analysis, respectively. The PB samples were collected in EDTA-K₂ anticoagulant tubes (≥2 mL) for lymphocyte subset analysis using anticoagulated whole blood.

### Measurement of cytokine and lymphocyte profiles

The concentrations of CPs in BALF, LPs in BALF, and LPs in PB were analyzed using multiparameter flow cytometry on the DxFLEX (Beckman Coulter Life Sciences, Indianapolis, IN, USA). The human custom 14-cytokine kit, which measures IL-1β, IL-2, IL-4, IL-5, IL-6, IL-8, IL-10, IL-12p70, IL-17A, IL-17F, IL-22, TNF-α, TNF-*β*, and IFN-*γ*, was purchased from Quantobio Biotechnology (Beijing, China), and assays were conducted according to the manufacturer's protocol. All BALF samples were analyzed using bead-based multiplex flow cytometry and were diluted 1:3 prior to testing. LPs, including CD3+, CD3 + CD4+, CD3 + CD8+, CD19+, and CD16 + CD56 + in both BALF and PB, were identified using a kit from Quantobio Biotechnology, which contained fluorescently labeled antibodies: CD3-FITC, CD16 + 56-PE, CD45-PerCP-Cy5.5, CD4-PC7, CD19-APC, and CD8-APC-Cy7. All samples, along with the manufacturer's quality control products, were immediately tested to ensure the accuracy of the detection. Data analysis was subsequently performed using Kaluza software (V2.1.1).

### Based on LASSO analysis and logistic regression model for discriminating Lym + LRTIs and Lym + NPIs

Baseline CRP, PCT, and serum amyloid A (SAA) were used to develop the Base logistic regression (LR) models, while 24 CP and LP features were evaluated for the BALF models. All variables were initially eligible for selection using least absolute shrinkage and selection operator (LASSO) within five-fold cross-validation. In each iteration, four folds formed the training set and the remaining fold served as the test set. To avoid information leakage, imputation parameters, feature selection, and model fitting were derived exclusively from the training data before being applied to the held-out fold. The regularization parameter *λ* was determined using the one-standard-error rule, and features selected in at least 80% of the folds were retained for final modeling. Fold-specific receiver operating characteristic curves and calibration plots were averaged across iterations. Discrimination and calibration were assessed separately in the training and test sets using the area under the curve (AUC) and Brier score, with 95% confidence intervals. Additional performance measures included accuracy, sensitivity, specificity, positive and negative predictive values, F1 score, and Cohen's kappa. Finally, the retained predictors were refitted using the full dataset to derive the Base and BALF models, expressed as

$$\mathrm{logit}(P)=\beta_{0}+\beta_{1}X_{1}+\cdots +\beta_{k}X_{k}.$$

### Comparison and bootstrap optimism correction of the BALF and Base models

The Base and BALF models were compared using differences in AUC (*Δ*AUC), with statistical significance assessed by the paired DeLong test. Improvements in risk prediction were further evaluated using the net reclassification improvement (NRI) and integrated discrimination improvement (IDI), with 95% CIs. Bootstrap internal validation with 1,000 resamples was used to estimate and correct for optimism. In each bootstrap sample, which was the same size as the original dataset and drawn with replacement, the complete modeling pipeline—including missing-data handling, feature selection, and logistic regression fitting—was repeated. Each fitted model was evaluated in both the bootstrap sample and the original dataset. For the *b*th iteration, optimism in the AUC was calculated as

$$\mathrm{Optimis}m_{b}=\mathrm{AU}C_{\mathrm{bootstrap},b}-\mathrm{AU}C_{\mathrm{original},b}.$$

The optimism-corrected AUC was then calculated as

$$\mathrm{AU}C_{\mathrm{corrected}}=\mathrm{AU}C_{\mathrm{apparent}}-\frac{1}{1000}\overset{1000}{\underset{b=1}{\sum }}\mathrm{Optimis}m_{b}.$$

Calibration intercepts and slopes were corrected analogously.

### Assessment of differences in cytokine and lymphocyte profiles according to lymphoma status and infection type (viral-only vs. bacterial-only)

Univariate analysis was performed to compare CPs and LPs between the Lym + LRTIs and non-Lym + LRTIs cohorts and between viral-only and bacterial-only infections. Four subgroup comparisons were conducted: Lym + viral-only LRTIs vs. Lym + bacterial-only LRTIs, Lym + viral-only LRTIs vs. non-Lym + viral-only LRTIs, Lym + bacterial-only LRTIs vs. non-Lym + bacterial-only LRTIs, and non-Lym + viral-only LRTIs vs. non-Lym + bacterial-only LRTIs. Etiology was classified at the patient level. Two pulmonologists independently reviewed microbiological results, clinical features, chest imaging, pathogen burden, potential copathogens, and treatment response while blinded to CPs and LPs results. Organisms were categorized as causative, possibly causative, colonization, contamination, or viral shedding/reactivation; disagreements were adjudicated by a microbiologist. Viral-only LRTIs required a causative virus without causative bacterial, fungal, *Mycoplasma*, or *Chlamydia* pathogens; bacterial-only LRTIs were defined similarly. Multiple causative organisms within one pathogen class remained in that category, whereas organisms from at least two classes indicated mixed infection. CMV or EBV detected by PCR or mNGS was considered causative only with compatible clinical and radiographic findings; otherwise, it was classified as reactivation or asymptomatic shedding and excluded from the viral category.

### Statistical analysis

The normality of continuous variables was assessed using the Shapiro–Wilk test. Continuous variables were summarized as medians with interquartile ranges (IQRs) and compared using the Mann–Whitney U test. Categorical data were presented as numbers (%), and were analyzed using either the chi-square test or Fisher's exact test. Statistical significance was defined as a two-sided *P* value less than 0.05. The statistical calculations and chart creation were performed using GraphPad Prism version 10 and Python version 3.13. The study workflow is presented in Figure 1, and the study was reported in accordance with the TRIPOD guidelines (13).

**Figure 1:**
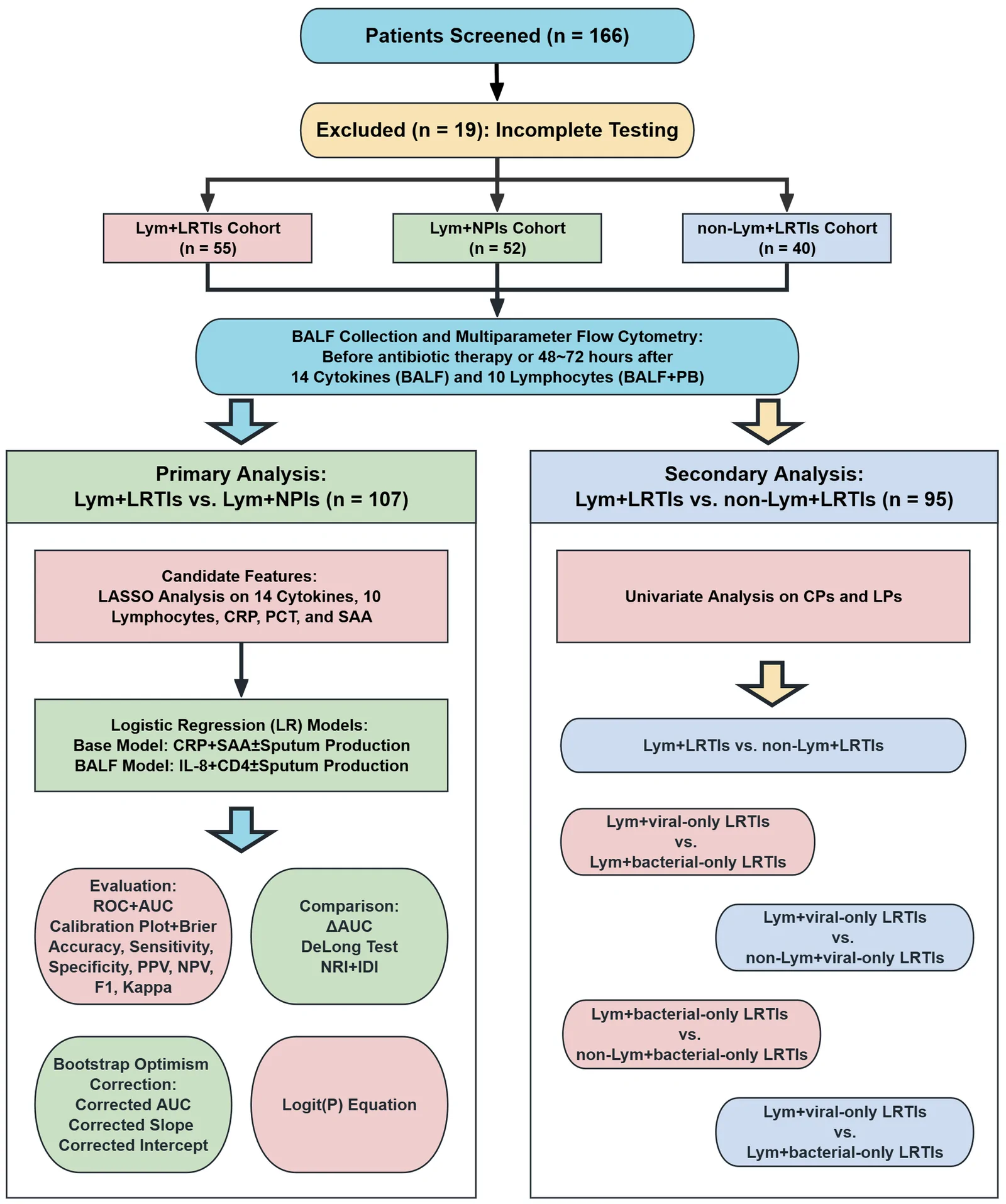
Flow chart of this study. This study initially enrolled 166 participants, of which 19 were excluded for failing to complete 14 cytokines and 10 lymphocytes testing. Finally, 147 cases were included in the study, consisting of 55 Lym + LRTIs, 52 Lym + NPIs, and 40 non-Lym + LRTIs. The primary analysis compared Lym + LRTIs with Lym + NPIs. Candidate features were selected using LASSO. Logistic regression (LR) models were subsequently developed and validated using five-fold cross-validation, including Base models (CRP and SAA, with or without sputum production) and BALF models. Model performance was evaluated using ROC curves with AUC, calibration plots with Brier score, accuracy, sensitivity, specificity, PPV, NPV, F1 score, and Kappa. Model comparisons were performed using *Δ*AUC with the DeLong test, NRI, and IDI. Bootstrap optimism correction was applied to estimate the optimism-corrected AUC, calibration slope, and calibration intercept. The secondary analysis compared Lym + LRTIs with non-Lym + LRTIs using univariate analysis of CPs and LPs, followed by subgroup comparisons: viral-only vs. bacterial-only LRTIs within each lymphoma-status group, and Lym + vs. non-Lym + patients within each infection category.

## Results

### Differential baseline characteristics across the three cohorts

A total of 147 participants were enrolled between January 1, 2022, and April 30, 2023, at the Aerospace Center Hospital. These patients comprised 55 lymphoma patients with confirmed LRTIs, 52 lymphoma patients who underwent bronchoscopy for pulmonary infiltrates and were adjudicated as having NPIs after comprehensive evaluation, including clinical presentation, chest imaging, and BALF microbiological testing, and 40 non-lymphoma patients with confirmed LRTIs. Table 1 illustrated the baseline of the participants, comparing two groups: Lym + LRTIs vs. Lym + NPIs cohorts, and Lym + LRTIs vs. non-Lym + LRTIs cohorts. The Lym + LRTIs cohort had a significantly higher incidence of sputum production (SP) compared to the Lym + NPIs cohort (*P* < 0.001). Additionally, these two cohorts differed significantly in lymphoma types (*P* < 0.01). Regarding laboratory findings, the Lym + LRTIs cohort had higher levels of white blood cell count, neutrophil percentage, CRP, PCT, SAA, and 1,3-beta-D-glucan compared to the Lym + NPIs cohort (*P* < 0.05). While the level of lymphocyte percentage was lower than that in Lym + NPIs cohort (*P* < 0.001). When comparing the Lym + LRTIs cohort to the non-Lym + LRTIs cohort, the former showed lower white blood cell counts and higher monocyte percentages (*P* < 0.001).

**Table 1:** Baseline characteristics of 147 subjects in the three cohorts.

Variables	Lym + LRTIs (*n* = 55)	Lym + NPIs (*n* = 52)	non-Lym + LRTIs (*n* = 40)	*P1* value	*P2* value
Age, years	59 (48, 67)	60 (37, 67)	62 (55, 69)	0.418	0.163
Male gender	32 (58.2%)	30 (57.7%)	24 (60.0%)	0.959	0.859
Body Mass Index, kg/m^2^	22.8 (21.3, 25.6)	23.1 (21.2, 25.4)	24.3 (21.7, 26.1)	0.796	0.213
Smoking	23 (41.8%)	16 (30.8%)	16 (40.0%)	0.235	0.859
Prior antibiotic use	23 (41.8%)	20 (38.5%)	21 (52.5%)	0.723	0.303
Symptoms					
Fever	22 (40.0%)	15 (28.8%)	16 (40.0%)	0.225	>0.999
Cough	43 (78.2%)	42 (80.8%)	35 (87.5%)	0.741	0.242
Sputum production	33 (60.0%)	14 (26.9%)	28 (70.0%)	<0.001	0.315
Dyspnea	21 (38.2%)	14 (26.9%)	22 (55.0%)	0.215	0.104
Comorbidity					
Chronic obstructive pulmonary disease	6 (10.9%)	4 (7.7%)	6 (15.0%)	0.568	0.553
Asthma	0 (0)	1 (1.9%)	2 (5.0%)	0.486	0.094
Hypertension	14 (25.5%)	11 (21.2%)	14 (35.0%)	0.599	0.314
Type 2 diabetes mellitus	12 (21.8%)	6 (11.5%)	11 (27.5%)	0.155	0.523
Coronary artery disease	9 (16.4%)	6 (11.5%)	7 (17.5%)	0.472	0.884
Cerebrovascular disease	3 (5.5%)	2 (3.8%)	7 (17.5%)	0.694	0.059
Chronic liver disease	6 (10.9%)	3 (5.8%)	2 (5.0%)	0.338	0.306
Chronic kidney disease	3 (5.5%)	1 (1.9%)	3 (7.5%)	0.336	0.686
Lymphoma type				0.004	-
HL	0 (0)	7 (13.5%)	-		
B-NHL	40 (72.7%)	23 (44.2%)	-		
T-NHL	6 (10.9%)	6 (11.5%)	-		
Unclassified-NHL	9 (16.4%)	16 (30.8%)	-		
Antineoplastic				0.257	-
Chemotherapy	11 (20.0%)	18 (34.6%)	-		
Rituximab/Obinutuzumab + chemotherapy	37 (67.3%)	32 (61.5%)	-		
BTK inhibitor+Rituximab/Obinutuzumab + chemotherapy	3 (5.5%)	1 (1.9%)	-		
Rituximab/Obinutuzumab + chemotherapy + CAR-T	2 (3.6%)	1 (1.9%)	-		
ADCT-402	2 (3.6%)	0 (0)			
Post-chemotherapy myelosuppression	16 (29.1%)	10 (19.2%)	-	0.235	-
LRTIs clinical score					
CURB-65 score				-	0.367
0 grading (score=0,1)	38 (69.1%)	-	22 (55.0%)		
1 grading (score=2)	8 (14.5%)	-	9 (22.5%)		
2 grading (score=3,4,5)	9 (16.4%)	-	9 (22.5%)		
CPIS				-	0.222
<=6	52 (94.5%)	-	35 (87.5%)		
> 6	3 (5.5%)	-	5 (12.5%)		
PSI				-	0.112
Ⅰ grading	0 (0)	-	3 (7.5%)		
Ⅱ grading	4 (7.3%)	-	7 (17.5%)		
Ⅲ grading	14 (25.5%)		10 (25.0%)		
Ⅳ grading	26 (47.3%)		15 (37.5%)		
Ⅴ grading	11 (20.0%)	-	5 (12.5%)		
Peripheral blood laboratory findings					
White blood cell count, cells/*μ*L	6.19 (4.38, 9.27)	4.92 (3.89, 6.30)	8.63 (7.01, 11.32)	0.018	<0.001
Neutrophil percentage, %	76.7 (68.1, 81.4)	64.5 (59.0, 66.9)	77.1 (74.9, 84.3)	<0.001	0.078
Lymphocyte percentage, %	12.0 (8.7, 17.3)	23.6 (19.1, 28.6)	12.7 (8.5, 15.3)	<0.001	0.982
Monocyte percentage, %	10.2 (7.0, 12.7)	8.6 (6.3, 11.5)	6.5 (4.3, 8.4)	0.259	<0.001
C-reactive protein (mg/L)	22.2 (12.8, 56.9)	2.0 (1.0, 3.4)	36.2 (15.3, 103.0)	<0.001	0.053
Procalcitonin (ng/mL)	0.10 (0.05, 0.16)	0.03 (0.02, 0.06)	0.13 (0.05, 0.81)	<0.001	0.138
Serum amyloid A (mg/L)	52.5 (12.5, 125.0)	2.6 (1.8, 3.5)	113.6 (23.8, 187.2)	<0.001	0.083
1,3-beta-D-glucan (G test)				0.010	0.870
Positive	5 (9.1%)	0 (0)	3 (7.5%)		
Weakly-reactive	4 (7.3%)	0 (0)	4 (10.0%)		
Negative	46 (83.6%)	52 (100%)	33 (82.5%)		

Data are presented as median (IQR), or *n* (%). *P1* values comparing Lym + LRTIs and Lym + NPIs cohorts while *P2* comparing Lym + LRTIs and non-Lym + LRTIs cohorts from two-tailed *χ^2^* test, two-tailed Fisher's exact test, or two-tailed Mann–Whitney *U* test. Lym, lymphoma; LRTIs, lower respiratory tract infections; NPIs, noninfectious pulmonary infiltrates; HL, Hodgkin's lymphoma; NHL, non-Hodgkin's lymphoma; BTK, Bruton's tyrosine kinase; CAR-T, chimeric antigen receptor T-cell immunotherapy; CPIS, clinical pulmonary infection score; PSI, pneumonia severity index.

### Candidate features and logistic regression models for differentiating LRTIs from NPIs in patients with lymphoma

In the five-fold cross-validation comparing the Lym + LRTIs and Lym + NPIs cohorts, missing values for CRP, PCT, and SAA were imputed using the median calculated from the corresponding training fold, and the same median was subsequently applied to the held-out test fold. LASSO analysis selected BALF_IL-8, CRP, and SAA in all five iterations, BALF_CD3 + CD4 + in four iterations, and BALF_IL-22 in three iterations; BALF_IL-5, BALF_IFN-*γ*, BALF_CD3 + CD8+, PB_CD3 + CD4+, and PB_CD3 + CD8 + were each selected twice, while BALF_IL-4, BALF_IL-12p70, BALF_IL-17F, BALF_CD3+, PB_CD16 + CD56+, and PCT were each selected only once, and the remaining features were never selected (Figure 2A). Ultimately, BALF_IL-8 and BALF_CD3 + CD4 + were retained as candidate features for the BALF models, whereas CRP and SAA were retained as candidate features for the Base models. Accordingly, four LR models were developed: BALF-A, which included BALF_IL-8 and BALF_CD3 + CD4+; and BALF-B, which incorporated sputum production (a clinical symptom that showed a statistically significant association) in addition to the BALF-A features; Base-A, which included CRP and SAA; Base-B, which incorporated SP in addition to the Base-A features. The four LR models demonstrated good discriminatory performance, with test-set AUCs ranging from 0.845 to 0.973; however, the Base models showed superior overall performance (Figure 2B). The calibration plots generally aligned closely with the ideal line (y = x), and the test-set Brier scores ranged from 0.053 to 0.167, with the Base models again demonstrating better performance (Figure 2B). Based on the test-set performance metrics, BALF-B slightly outperformed BALF-A, achieving a higher AUC (0.853 vs. 0.845), a lower Brier score (0.151 vs. 0.167), and higher accuracy (0.804 vs. 0.766) and F1 score (0.802 vs. 0.787) (Figure 2C). Among the Base models, Base-A achieved a slightly higher test-set AUC (0.973 vs. 0.969) and a lower Brier score (0.053 vs. 0.059) than Base-B. Base-A also yielded higher accuracy (0.943 vs. 0.905) and F1 score (0.947 vs. 0.903) (Figure 2C). The final equations for four LR models were as follows (Figure 2D),

logit(BALF_A)=−0.971+0.422×IL_8/100−0.029×CD4;

logit(BALF_B)=−1.246+0.415×IL_8/100−0.033×CD4+1.180×SP;

logit(Base_A)=−7.271+0.711×CRP+0.583×SAA;

logit(Base_B)=−7.424+0.718×CRP+0.559×SAA+0.577×SP.

**Figure 2:**
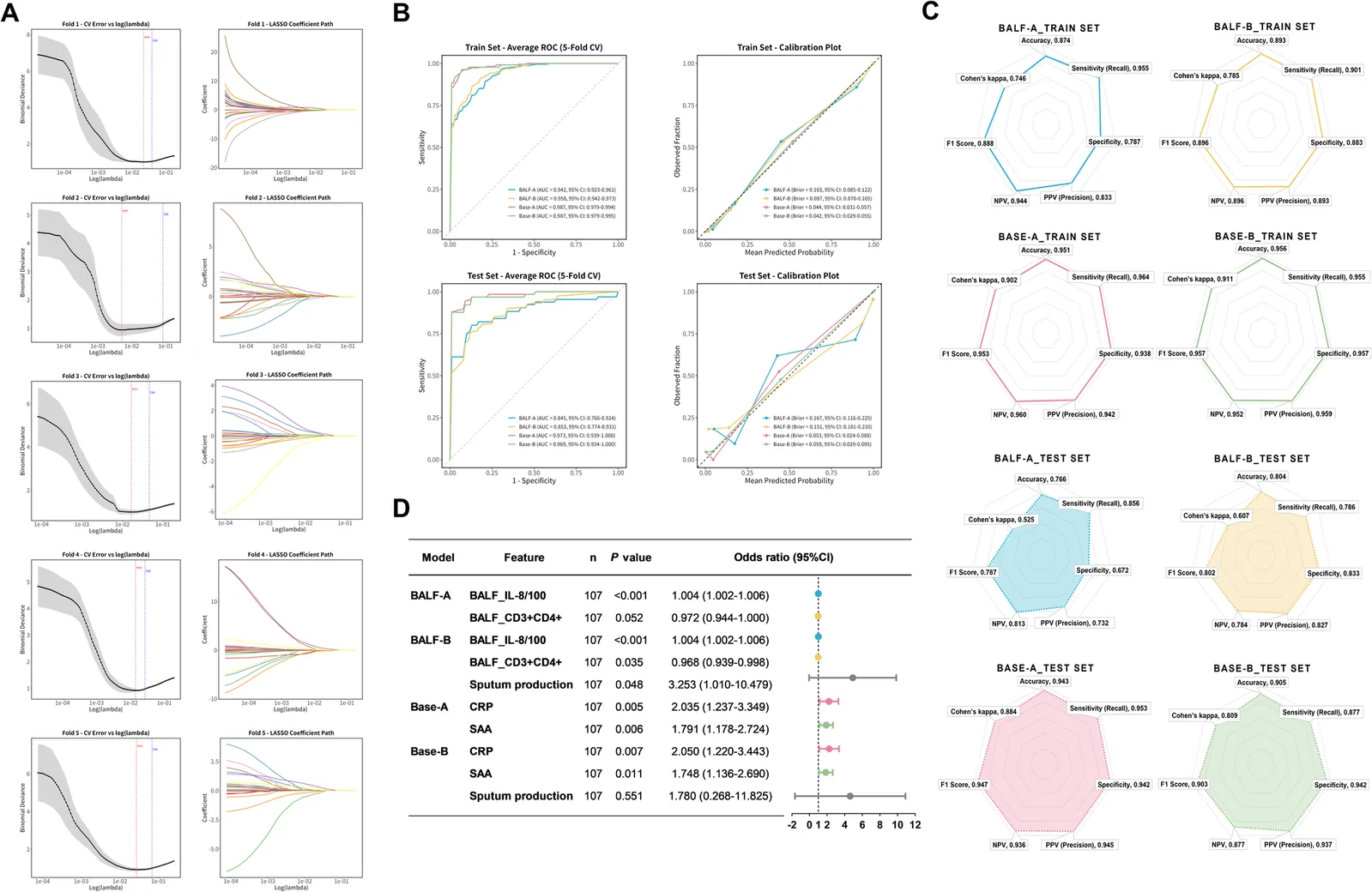
Discriminate Lym + LRTIs and Lym + NPIs through BALF and Base logistic regression models. **(A)** Cross-validated binomial deviance and LASSO coefficient paths across Folds 1-5 for 14 cytokines and 10 lymphocytes. The red and blue dashed lines indicate *λ*_min and *λ*_1SE, respectively. Predictors with nonzero coefficients at the fold-specific *λ*_1SE were selected. **(B)** ROC curves with AUC and calibration plots with Brier score of the four logistic regression models in both train and test sets. **(C)** The performance metrics included accuracy, sensitivity (recall), specificity, positive predictive value (PPV; precision), negative predictive value (NPV), F1 score, and Cohen's kappa of the BALF-A_Train Set, BALF-B_Train Set, Base-A_Train Set, Base-B_Train Set, BALF-A_Test Set, BALF-B_Test Set, Base-A_Test Set, Base-B_Test Set, respectively. **(D)** Forest plots of equation parameters for the four logistic regression models.

### DeLong test and bootstrap optimism correction of the BALF and Base models

Adding SP to the BALF model did not significantly improve discrimination (*Δ*AUC = 0.008, *P* = 0.791); however, it yielded a significant improvement in risk reclassification (NRI = 0.666, 95% CI: 0.316∼0.976) and a borderline increase in the integrated discrimination improvement (IDI = 0.066, 95% CI: -0.004∼0.136) (Table 2). In contrast, adding SP to the Base model conferred no improvement (*Δ*AUC = −0.004, *P* = 0.167). Compared with Base-A, BALF-A had a significantly lower AUC (*Δ*AUC = −0.128, *P* < 0.001), together with negative NRI (−1.274, 95% CI: −1.536∼−0.987) and IDI (−0.341, 95% CI: −0.477∼−0.209). Similar results were observed for Base-B vs. BALF-B (*Δ*AUC = −0.117, *P* = 0.002; NRI=−0.973, 95% CI: −1.274∼−0.662; IDI=−0.263, 95% CI: −0.403∼−0.127) (Table 2). Bootstrap validation further supported the robustness of the LR models. After correction for optimism, the AUCs were 0.980 for Base-A and 0.977 for Base-B, compared with 0.872 for both BALF-A and BALF-B. The Base models also exhibited substantially lower optimism (0.006∼0.008 vs. 0.056∼0.057) and calibration slopes closer to unity (0.708∼0.773 vs. 0.500∼0.527) (Table 3).

**Table 2:** Comparison of the Base model and the BALF model using DeLong's test.

Model comparison	*Δ*AUC	DeLong's Test *P* value	NRI (95%CI)	IDI (95%CI)
BALF-A vs. BALF-B	0.008	0.791	0.666 (0.316∼0.976)	0.066 (−0.004∼0.136)
Base-A vs. Base-B	−0.004	0.167	0.239 (−0.091∼0.527)	−0.012 (−0.027∼0.001)
Base-A vs. BALF-A	−0.128	<0.001	−1.274 (−1.536∼-0.987)	−0.341 (−0.477∼−0.209)
Base-B vs. BALF-B	−0.117	0.002	−0.973 (−1.274∼−0.662)	−0.263 (−0.403∼−0.127)

BALF-A, IL-8 + CD4; BALF-B, IL-8 + CD4 + sputum production; Base-A, CRP + SAA; Base-B, CRP + SAA + sputum production. NRI, net reclassification improvement; IDI, integrated discrimination improvement.

**Table 3:** Optimism-corrected parameters via 1,000 bootstrap iterations.

Model	Apparent AUC	Optimism	Corrected AUC	Corrected calibration slope	Corrected calibration intercept
BALF-A	0.928	0.056	0.872	0.527	−0.150
BALF-B	0.929	0.057	0.872	0.500	−0.137
Base-A	0.986	0.006	0.980	0.773	−0.042
Base-B	0.985	0.008	0.977	0.708	−0.112

BALF-A, IL-8 + CD4; BALF-B, IL-8 + CD4 + sputum production; Base-A, CRP + SAA; Base-B, CRP + SAA + sputum production.

### Differential cytokine and lymphocyte profiles according to lymphoma status and infection type (viral-only vs. bacterial-only)

A total of 133 patient-pathogen detections were recorded among 95 patients with LRTIs, with the top five being *SARS-CoV-2*, MP, EBV, and *Pneumocystis jirovecii*, with CMV, *Acinetobacter baumannii*, and *Pseudomonas aeruginosa* tied for fifth place with equal case numbers. Notably, *SARS-CoV-2* was detected in 22 of the 95 LRTIs (23.2%), likely reflecting the enrollment period from January 2022 to April 2023, which overlapped with Omicron-era *SARS-CoV-2* circulation in China. Except for *SARS-CoV-2* and *A. baumannii*, the proportions of the other five pathogens were higher in the Lym + LRTIs cohort compared to the non-Lym + LRTIs cohort (Figure 3A). Nearly thirty percent of LRTIs cases were attributable solely to viral infections, with bacterial infections representing the second most common etiology (Figure 3A). We performed four comparisons across four subgroups: Lym + viral-only LRTIs (*n* = 19), Lym + bacterial-only LRTIs (*n* = 11), non-Lym + viral-only LRTIs (*n* = 8), and non-Lym + bacterial-only LRTIs (*n* = 13) (Figure 3B). PB_CD3 + levels differed significantly between Lym + viral-only and Lym + bacterial-only LRTIs (*P* = 0.018). Between Lym + and non-Lym + patients with viral-only LRTIs, BALF_IL-5 (*P* = 0.035), BALF_IL-17A (*P* = 0.033), BALF_CD19+ (*P* = 0.039), and PB_CD19+ (*P* = 0.005) levels differed significantly. Likewise, between Lym + and non-Lym + patients with bacterial-only LRTIs, BALF_IL-8 (*P* = 0.018), BALF_CD3+ (*P* = 0.035), and BALF_CD19+ (*P* = 0.002) levels differed significantly. In the comparison between the full Lym + LRTIs and non-Lym + LRTIs cohorts (Figure 3B), eight parameters differed significantly: BALF_IL-6 (*P* = 0.001), BALF_IL-8 (*P* = 0.016), BALF_IFN-*γ* (*P* = 0.009), BALF_CD3+ (*P* < 0.001), BALF_CD3 + CD8+ (*P* = 0.035), BALF_CD19+ (*P* < 0.001), BALF_CD16 + CD56+ (*P* = 0.006), and PB_CD19+ (*P* < 0.001).

**Figure 3:**
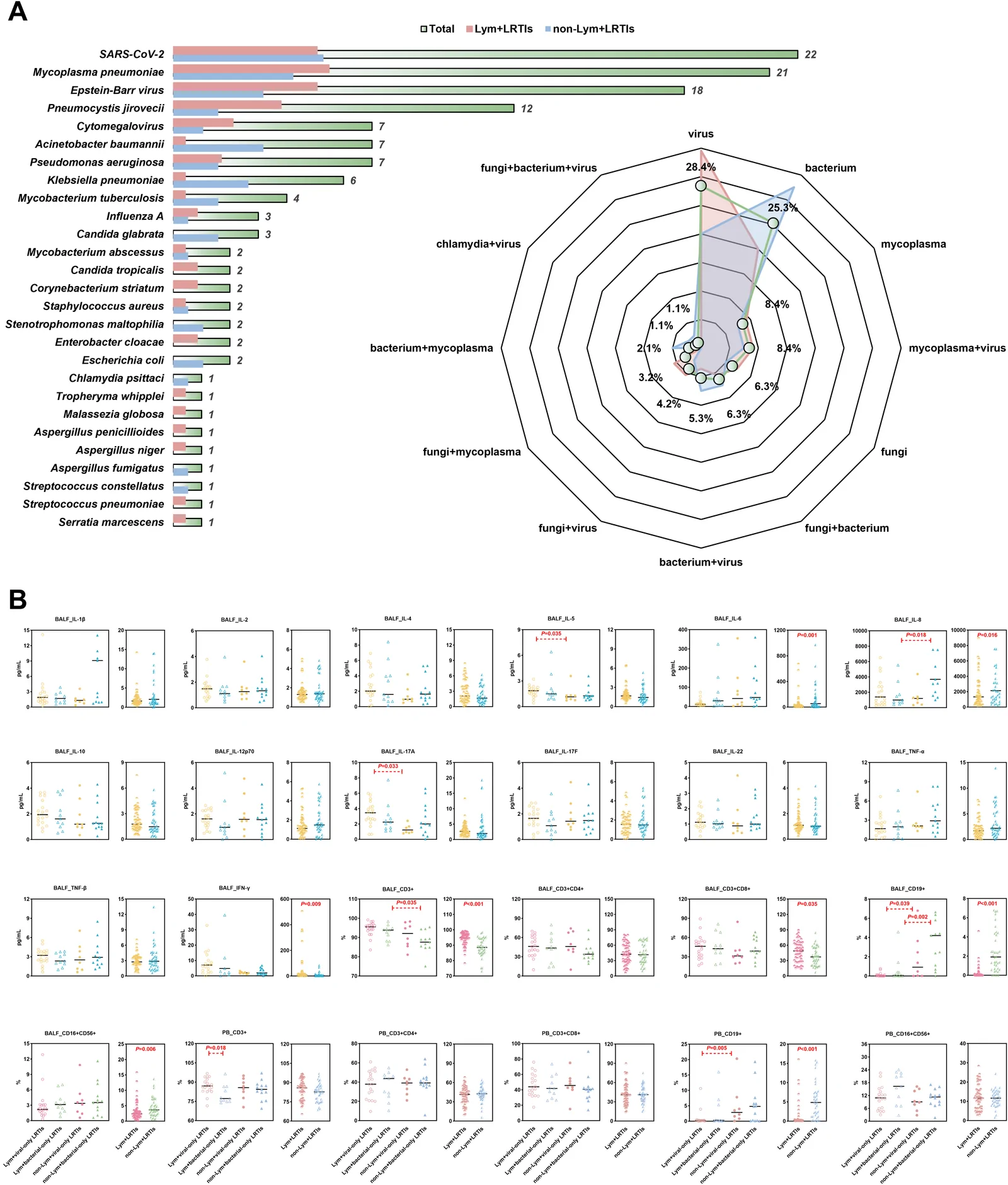
Pathogen spectrum and differential cytokine and lymphocyte profiles by infection type and lymphoma status in LRTIs cohorts. **(A)** The bar chart displays the 133 pathogens detected in 95 infected patients: green represents the overall distribution among all LRTIs cases, red represents the Lym + LRTIs cohort, and blue represents the non-Lym + LRTIs cohort. The radar chart displays the proportion of 12 infection types in the 95 LRTIs cases: green represents the overall distribution, red represents the Lym + LRTIs cohort, and blue represents the non-Lym + LRTIs cohort. **(B)** Box plots with median lines for 14 cytokines and 10 lymphocytes. Left panels: comparisons across four infection-type subgroups: Lym + viral-only LRTIs (*n* = 19), Lym + bacterial-only LRTIs (*n* = 11), non-Lym + viral-only LRTIs (*n* = 8), and non-Lym + bacterial-only LRTIs (*n* = 13). Subgroup assignment followed the etiological adjudication algorithm described in Methods; only the virus-only and bacterium-only strata were included in the four infection-type comparisons. Right panels: comparisons between the full Lym + LRTIs and non-Lym + LRTIs cohorts.

## Discussion

In this retrospective study of 147 patients, we developed and internally validated LR models based on BALF CPs and LPs to distinguish LRTIs from NPIs in patients with lymphoma. Several findings warrant emphasis. First, a parsimonious BALF model comprising IL-8, CD3 + CD4+ T cells, and sputum production achieved good discrimination, with a test-set AUC of 0.853 and an optimism-corrected AUC of 0.872 after 1,000-bootstrap validation. Second, although adding sputum production to the BALF model did not significantly improve the AUC (*P* = 0.791), it significantly improved risk reclassification (NRI = 0.666, 95% CI: 0.316∼0.976). Third, models based on routine inflammatory markers (CRP and SAA) significantly outperformed the BALF models (optimism-corrected AUCs of 0.977∼0.980; DeLong *P* < 0.01) and were considerably more robust to overfitting.

The selection of IL-8 is biologically plausible. Previous research in patients with non-Hodgkin lymphoma showed that IL-8 and other cytokines were elevated during respiratory bacterial infections (2). Single-cell analyses have also linked increased pulmonary CXCL8 expression to neutrophil accumulation and lung injury in severe viral and bacterial pneumonia (11, 14). These findings support BALF IL-8 as a marker of local infectious inflammation. In the lower respiratory tract, alveolar macrophages are major producers of IL-8, although type II pneumocytes, fibroblasts, and endothelial cells also contribute (15). IL-8 promotes adhesion-molecule expression and recruits neutrophils—key defenders against bacterial and fungal pathogens—into infected lung tissue (16). Binding to CXCR1 and CXCR2 further activates neutrophil metabolic and morphological responses (17). These mechanisms may explain elevated BALF IL-8 levels in patients with LRTIs and underscore its role in pulmonary host defense. Conversely, higher BALF CD3 + CD4 + proportions were associated with lower odds of LRTIs (odds ratio 0.968∼0.972 per percentage point), suggesting that preserved local T-cell immunity protects against infection. This notion is consistent with the central role of CD4+ T cells in pulmonary host defense against bacterial, viral, and fungal pathogens (18) and, conversely, with the increased susceptibility to LRTIs imposed by lymphoma and antineoplastic treatment. One additional point requires separate clarification. Lymphoma subtype distributions differed between the Lym + LRTIs and Lym + NPIs cohorts (Table 1, *P* = 0.004). In a sensitivity analysis restricted to B-NHL, IL-8 remained significantly different between groups (*P* < 0.001), whereas BALF CD3 + CD4 + did not (*P* = 0.180). Thus, although the IL-8 signal was robust, the overall IL-8/CD4/sputum production model as a whole should be viewed as a classifier derived from a subtype-imbalanced cohort.

An important and perhaps unexpected finding was that the Base models significantly outperformed the BALF models. Several explanations should be considered. First, CRP and SAA are potent acute-phase reactants reflecting large, systemic responses, whereas BALF cytokines are local signals susceptible to lavage dilution, variable fluid recovery, and the focal distribution of infiltrates. Second, the BALF models showed greater overfitting (corrected calibration slopes 0.50∼0.53 vs. 0.71∼0.77 for the Base models); with only 107 patients, parsimonious two-variable models are inherently more stable. We therefore emphasize that the BALF model is not intended to replace routine inflammatory markers, which remain accurate, inexpensive, and universally available (19). Its potential value may instead lie in patients with atypical presentations or discordant clinical, radiological, microbiological, and systemic inflammatory findings (20).

Adding sputum production to the BALF model significantly improved the NRI but did not significantly increase the AUC, while the IDI improvement was borderline. This suggests that sputum production may alter the ranking of individual predictions without materially improving overall discrimination. NRI findings should be interpreted cautiously because they are sensitive to model calibration and the definition of clinically meaningful risk categories (21). Conversely, sputum production conferred no benefit to the Base model, possibly because its diagnostic information was already captured by CRP and SAA. Future studies should evaluate incremental predictors using prespecified probability thresholds and decision-curve analysis.

The secondary analysis revealed differences in BALF IL-6, IL-8, IFN-*γ*, and several T-, B-, and NK-cell subsets between patients with and without lymphoma who had LRTIs, as well as infection type-specific variations. Collectively, these findings suggest that lymphoma and its treatment may reshape the pulmonary immune milieu during infection (14). Nevertheless, the results should be considered exploratory because the viral-only and bacterial-only subgroups were small and multiple immune parameters were tested without adjustment for multiple comparisons. *SARS-CoV-2* was the most frequently detected pathogen, accounting for 23.2% of LRTIs, consistent with enrollment overlapping the Omicron era in China. This temporal context should be considered when generalizing our findings to other periods and settings.

Several limitations should be acknowledged. This retrospective, single-center study included a modest number of patients who underwent bronchoscopy, potentially introducing selection bias. Internal validation cannot replace independent prospective validation. Moreover, heterogeneity in lymphoma subtype and treatment may have confounded the observed BALF associations, while variations in BALF dilution, sampling site, and timing relative to antimicrobial therapy may have influenced biomarker concentrations.

## Conclusion

A BALF cytokine-lymphocyte profile-based model incorporating IL-8, CD3 + CD4+, and sputum production accurately distinguished LRTIs from NPIs in patients with lymphoma, although the model based on routine inflammatory markers (CRP and SAA) performed significantly better. BALF immune profiling may therefore complement conventional biomarkers and reveal distinct local immune signatures associated with lymphoma status and infection type. External validation is required before clinical implementation.

## Data Availability

The original contributions presented in the study are included in the article/Supplementary Material, further inquiries can be directed to the corresponding authors.
