# Supplementary material for: Diagnostic value of bronchoalveolar lavage fluid cytokine and lymphocyte profiles for lower respiratory tract infections in patients with lymphoma
Source: Front Med (Lausanne). 2026 Sep 23;13:1904717. doi: 10.3389/fmed.2026.1904717 (PMC13642284; doi:10.3389/fmed.2026.1904717)
Supplement: Supplementary file 1 [file Table1.docx]

**TABLE S1. Criteria Derived from the Chinese Thoracic Society (CTS) Guidelines on the Diagnosis of LRTIs**

**Clinical definition: meeting one or two of the following**

**1.Signs/Symptoms:**

• New onset/worsening cough, dyspnea, tachypnea, or new purulence or change in sputum character

• Fever

• Rales or bronchial breath sounds

• WBC >10×10^9^/L or < 4×10^9^/L

**2. Imaging test evidence:**

• Two or more serial chest imaging test results with at least one of the following: new or progressive infiltrate, consolidation, or cavitation

**Laboratory definition: meeting the clinically definition and at least one of the following**

• Organism identified from blood unrelated to another source

• Organism identified from pleural fluid

• Positive culture from minimally-contaminated LRT specimen or lung tissue

• Histopathologic exam shows an abscess formation, intense polymorphonuclear accumulation, or fungal hyphae or pseudohyphae

• Identified viral, *Legionella*, and other atypical pathogens from respiratory secretions or tissue by non-culture testing method

• Positive antibody or antigen testing of viral or atypical pathogens in sera or urine

**WBC, white blood cell. LRT, lower respiratory tract. LRTIs, lower respiratory tract infections.**
